# The role of navigation services in supporting mental health and addictions care transitions: A qualitative exploration of perspectives from transitional-aged youth, family, and service providers (part 2)

**DOI:** 10.1016/j.hctj.2024.100082

**Published:** 2024-11-23

**Authors:** Roula Markoulakis, Hinaya Cader, Karen Wong, Sugy Kodeeswaran, Tracey Addison, Cathy Walsh, Jocelyn Charles, Amy Cheung, Deepy Sur, David Willis, Anthony Levitt

**Affiliations:** a Sunnybrook Research Institute, Canada; b University of Toronto, Canada; c Sunnybrook Health Sciences Centre, Canada; d Family Advisory Council, Family Navigation Project, Sunnybrook Health Sciences Centre, Canada; e Ontario Association of Social Work (Present Affiliation: Ontario College of Family Physicians), Canada; f Strides Toronto (Present Affiliation: Keystone Child, Youth, and Family Services), Canada

**Keywords:** Transitional-aged youth, Caregivers, Families, Service providers, Mental health, Addictions, Transitions in care, Continuity of care, Mental health services, Patient navigation, Navigation services

## Abstract

**Introduction:**

Transitional-aged youth (TAY) are at a vulnerable stage of their development in which mental health and/or addiction (MHA) issues tend to manifest and/or increase in severity. These youth also tend to find themselves caught in the gap between child and adult MHA services, often resulting in sub-optimal access to and transition through MHA services. Navigation services may be one way to close this and other system gaps and improve service utilization and supports for TAY. The objective of this study was to explore the perspectives of TAY, family members, and system providers regarding the support that can be provided by navigation services in addressing the needs and barriers encountered during transitions in MHA care.

**Methods:**

This is a descriptive qualitative study of TAY, family, and provider perspectives on the role of navigation as it pertains to transitions in care for TAY with MHA concerns. Focus groups and semi-structured interviews were conducted with 63 participants with varying levels of familiarity with navigation. Participants were asked about their experiences with navigating transitions through the MHA system and their views on the role of navigation services in supporting transitions in care. Data was analyzed utilizing a thematic analysis approach.

**Results:**

Five themes emerged during data analysis: navigation to traverse difficult pathways, navigation to ensure appropriate and comprehensive care, navigation to sustain continuity of care, navigation to support informed care, and navigation to facilitate TAY and family involvement.

**Discussion:**

These findings contribute to an understanding of how navigation services can be meaningful in mitigating the challenges faced by TAY and their families when seeking help for MHA issues. Navigation services have the potential to support MHA system transformation for enhanced transitions in care for TAY with MHA concerns and their families.

## 1 Introduction

Transitional-aged youth (TAY; typically considered to be 12–29 years old), experience a multitude of mental health and/or addictions (MHA) care transitions and subsequent needs and barriers when accessing and transitioning through needed supports.^1^ TAY encounter a multitude of transitions in their care trajectories, including, but not limited to, hospital or live in treatment to community, transitions between providers, transitions between public and private care systems, and transitions between child and adult care systems – the latter receiving the bulk of attention in the literature.2., 3. Unfortunately, TAY are often not adequately prepared for or supported through care transitions, often attributed to resource constraints, lack of communication and collaboration between providers, and lack of information about upcoming transitions and care options.^4^ Without effective transitions in care, TAY are at risk of discontinuity or interruptions to care, or of disengaging from care entirely.2., 5. For these reasons, the transitional age is a critical target for ensuring continued care where needed, to support appropriate MHA care into adulthood. A recent scoping review identified the importance of proactive preparation for transitions, along with holistic supports, collaborative relationships, and empowering TAY and families, in order to enable effective transitions in MHA care.^4^ Furthermore, the companion paper for the present study identified themes of pathways to care (including subthemes of timeliness of help-seeking, accessibility of care, and clarity of care pathways), appropriate and comprehensive care (including appropriate care, flexible care, comprehensive support, and skills and approaches of providers), continuity of care (including uninterrupted care, continuous supports, and focus on transition preparation and readiness), informed care (including access to resource information, guidance, and choice), family involvement (including practical support, soft support, caregiver strain, and confidentiality), and TAY involvement (including participation in care, TAY-friendly care, and independence) as key barriers and facilitators in TAY MHA care transitions.^6^

Numerous recommendations exist for services to support TAY in these ways. Adding training and increasing responsibility for transitions within existing services is one possibility; however, would require placing responsibilities on services that already face competing demands.^7^ Creating new services designated for this age group is another possibility which would offer clarity for TAY and families, yet would also in effect add to the number of transitions experienced by TAY; and the designated services themselves would be inundated with referrals from both child and adult sectors.^7^ Although all proposals have their merits, the frequent threats and interruptions to continuous care^8^ experienced by TAY with MHA concerns necessitate a proactive approach^9^ that prevents these disruptions and “holds” the family in times when continuity has been broken. Services that function as a “bridge” to support TAY and families across systems and levels of care and support them in finding their way through their care can be an effective approach for ensuring TAY with MHA concerns and their families find and access the care that they need. The need for support for patients and families in navigating the health care system has been identified as a means of improving access to care.^10^ Moreover, access to and navigation of services has been identified as a core challenge facing TAY in particular. Similarly, the Mental Health Commission of Canada identified a need for navigators trained specifically for TAY and views navigation as an important component to a tiered approach that addresses the range of needed services for the MHA needs of these youth (i.e., universal health promotion through to specialized inpatient/residential treatment settings).^11^ Navigation services are thus an important intermediary for service integration and can be a core component of an efficient, integrated system that effectively supports transitions in care.

Navigation was developed as a clinical service to help patients address care disparities that arise as a result of complex care systems laden with financial, communication, information, systemic, and emotional barriers.^12^ Navigators provide patient-centered service, guiding individuals through their care plans and reducing barriers to timely access and transition.^12^ While there are multitudes of services available in the MHA system, pathways to and through the most appropriate care are unclear. Navigation services in MHA care are focused on reducing barriers to care, providing client-centered support, and integration of care.^13^ As such, navigation holds the potential to be an essential support in the MHA system.^14^ Many Navigation programs have emerged to help patients find their way through the siloes of the MHA system by connecting them to appropriate supports, and empowering them in managing their health.^15^ Although there have been efforts made to explore TAY and their families’ needs with regard to accessing and transitioning through MHA care, these needs have not been explored in relation to proposed system solutions with perspectives from various levels within the system. Given the recently accelerating implementation of navigation supports in the MHA system, there is a need to understand the perspectives of TAY with MHA concerns, family members, and service providers on the utility of navigation to address the needs of TAY and their families.

## 2 Methods

This study utilized qualitative descriptive methods to explore TAY, family, and service provider perspectives on the ways in which navigation services can support the needs of TAY and families in effectively accessing and transitioning through MHA care.^16^ Qualitative description is indicated where the aim is to generate straightforward, comprehensive summaries of phenomena in everyday terms.^16^ The data presented in this study are part of a larger overarching study exploring the perspectives of TAY, family members, and service providers regarding the supports needed by TAY and families when accessing and transitioning through MHA care (reported in the Part 1 companion paper to this piece).^6^ Although Canada has a socialized health care system, MHA services operate both privately and publically. Long waitlists for public services may drive TAY and families to private care,17., 18. creating disconnects that lead to care transitions. Furthermore, public MHA services are organized into child (under 18) and adult (over 18) sectors, also leading to transitions at this age.^19^ The research question guiding this particular study was: what are the perspectives of TAY, family members, and service providers regarding the support that can be provided by navigation services in addressing the needs of TAY and families when accessing and transitioning through MHA services?

All methodological procedures are described in Part 1.^6^ To summarize, ethics approval was obtained from the Sunnybrook Health Sciences Centre Research Ethics Board. The research team comprised representatives from local and provincial MHA services and associations. The team included researchers, service providers (e.g., psychiatrists, social workers, peer support workers), caregivers with lived experience, youth with lived experience, and decision-makers, who supported all phases of the research study and lent their perspectives to all procedures and findings outlined below. TAY, families, and service providers connected to various MHA services in the Greater Toronto Area and across the province (e.g., Family Navigation Project, East Metro Youth Services, Canadian Mental Health Association branches) were invited to participate via recruitment posters. Participants completed semi-structured one-on-one or focus group interviews asking about the needs of TAY and families with respect to transitions in MHA care and the role of navigation in supporting these needs. There were 63 participants in this study (14 TAY, 26 family, and 23 service providers). Of both TAY and family participants, half (n=20) had accessed navigation services (e.g., through the FNP or other services). Youth (71 % women) ranged in age from 17 to 29, with an average age of 23. Family members (88 % women) ranged in age from 48 to 61, with an average age of 54. Twelve service provider participants identified navigation as a key component of their work and/or were front-line staff at a navigation service. Other service provider roles included social workers, transitional youth workers, family support workers, intake workers, program managers/supervisors, and counsellors. Service providers (91 % women) ranged in age from 26 to 60, with an average age of 36. A total of 53 participants took part in individual interviews, while the remaining ten took part in one of three focus groups conducted. Participants were asked about their experiences in seeking and accessing MHA services, needs during transitions through MHA supports (reported in Part 1), and the role of navigation services in supporting MHA care transitions for TAY and families (reported in the present paper). Focus groups and interviews were recorded and transcribed, and data were analyzed thematically.^20^ Analysis was conducted by three coders (HC, KW, RM), who generated a preliminary codebook by coding nine of the same transcripts. Analysis was conducted using MAXQDA 2020. The codebook was refined as remaining transcripts were coded and used to guide coding of remaining transcripts, with regular meetings and discussion for documented refinement of the codebook and to maintain an audit trail. Codes were arranged into themes, and findings were validated with the broader team.

## 3 Results

Six themes emerged regarding the ways in which navigation supports access to and transitions through care: *navigation to traverse difficult pathways, navigation to ensure appropriate and comprehensive care, navigation to sustain continuity of care, navigation to support informed care,* and *navigation to facilitate TAY and family involvement.* As such, it was found that navigation services can support the TAY and family transition needs identified in part 1 of this study. Participant groupings are indicated next to the participant number (TAY, family (F), or service provider (SP)) following sample quotes.

### 3.1 Navigation to traverse difficult pathways

Navigation can support TAY and families in treading the often difficult pathways to MHA care identified in part 1 of this study. Navigation can simplify this process by saving TAY and family time and effort and connecting them to the right supports; streamlining care pathways to improve access, coordination, and transitions through services; and advocating for TAY and families in the complex MHA system.

Navigators may have the skills and expertise to locate the most appropriate supports based on TAY needs, in an efficient manner. One caregiver described how their navigator had connected the TAY with an addictions counsellor in the community, who proved to be a beneficial and meaningful support beyond MHA concerns:

“drawing from her own experience and her professional experience to help him cope, put in certain measures, how to move on. But not only to stay sober, she helps him navigate his life, his job, what to do in your spare time, and just helping him organize his life” (005-F).

Navigator support in connecting to appropriate care saves valuable TAY and family time and effort that would otherwise be expended trying to locate and access services themselves, especially in times of service transitions. Speaking to this, one caregiver shared, “*it really helps you find the right things instead of spinning your wheels and getting frustrated and feeling lost*” (019-F). Similarly, another caregiver indicated that their navigator “*went and did all that work with me and for me, and they’re experts at it*” (004-F). Participants shared that navigators also provide crucial support beyond identifying appropriate services: “*they also offer to help you connect with whatever service that is, even to make an appointment, or facilitate the referral, get information, pass on information. That was very helpful because those things can be challenging*” (003-F).

Navigation can support TAY and families in paving the care pathway specific to the TAY’s MHA needs. One navigator described navigation support in traversing the care pathway as “*getting somebody from one place to another. It’s not like a straight line. It’s a complex maze that you’re helping someone move through*” (026-SP). For caregivers, this “*was really like holding my hand through the process*” (008-F). One TAY participant expanded on this, stating that navigation was “*having someone there to help me and direct me in the direction I needed to go. Not necessarily holding my hand the entire way, but leading me in the right direction*” (006-TAY). Given the complex and multi-faceted transitions experienced by TAY and families in the MHA system, participants highlighted the expert information and guidance provided by navigators throughout the care trajectory as crucial in successful transitions. For example, in many instances, supporting TAY and families along their care pathways necessitates the use of creative solutions. One navigator indicated that they will support families when needed care is not immediately available, stating, “*we have to be incredibly creative about ways to get them something of their needs met [when] we know they can’t get therapy or they’re on a waitlist*” (026-SP). These creative solutions can involve a number of possible approaches, such as developing a care plan that includes interim supports for the TAY and the whole family, exploring and identifying services that are well-matched to TAY and family needs with a shorter waitlist but that are perhaps not available locally (then supporting through transportation considerations), psychoeducation and coping strategies, etc. Further to this, another navigator shared an example of how navigation can improve access, especially when a client faces health inequities:

“you’ve got families who have very different levels of disparity, but we have actually been successful with service providers doing a sliding scale who would never have done a sliding scale until we had a conversation with them” (015-SP).

Moreover, navigation supports coordination and transitions by breaking down the aforementioned care silos and encouraging communication between providers. A navigator described this role as follows:

“collaboration, being the ones to say these three people need to get on the phone together, and we’ll do the emailing of admins to make sure that there’s a time convenient to everyone. And usually those professionals want to have that call and they say, ‘I’m happy to get on the phone and collaborate,’ but no one’s doing the work and they don’t have the time” (028-SP).

Another navigator elaborated on the importance navigators place on relationship-building across the MHA system to support this collaboration. One navigator explained, *“[it’s] very much a collaborative stance. Even when we’re advocating for our clients to access services…knowing that engaging in a positive relationship with another professional will produce more than if you don’t develop that relationship*” (047-SP). Ultimately, these relationships support the advocacy work of navigators to help streamline TAY and families’ care transition pathways, as one navigator noted, “*there’s a lot more advocacy we can do together. So say they called a service like 10 times, they don’t hear back. And I’ll call and I’ll get a response*” (026-SP).

### 3.2 Navigation to ensure appropriate and comprehensive care

Recognizing the difficulties youth and families encounter in accessing specialized and well-matched services for their needs, Navigation helps ensure access to appropriate and comprehensive MHA care for TAY and families. Navigators perform thorough intake assessments, and utilize this information in tandem with extensive knowledge of the MHA system and nuances of available services to match TAY and families them to the most appropriate care for their needs.

By conducting thorough needs assessments, navigators are able to acquire a complete picture or story of what TAY and families are experiencing with regard to MHA care. These navigation needs assessments consider biopsychosocial factors alongside service preference, access, and coordination considerations, to inform the navigation plan and process. One navigator participant explained:

“gather information about what the youth has been experiencing, a little of their background, mental health background, educational background, personal background, family background, what they have tried, what was helpful, what wasn’t helpful, what their goals would be, so what would interest them. It’s really taking the time to learn about them and what they would feel would be most helpful for them” (045-SP).

Navigation programs operate flexibly, so that TAY and families are given the time they need to share their stories and experiences and to access support in a manner that suits their needs. Another navigator participant shared:

“there’s more flexibility around that so if someone needs to spend twenty, thirty or forty minutes with me, I’m able to do that…It’s really, I think, the meaning of patient-centred, that we’re molding our program around what fits the person, not some image of what we think the program should be” (048-SP).

Since navigators are able to acquire a holistic view of the experiences of TAY and families, they are better positioned to match TAY and families to the supports that meet their needs across multiple domains, and simplify the process of connecting to the right supports to help reduce mismatches to care and unnecessary care transitions that would follow:

“a navigator and navigation program can help the youth and family to connect with a person or a program that’s really fit for their needs and match for their needs, which means that there is a greater chance of success for what they are doing. Without somebody who is helping to do that, then a person can kind of jump around different programs” (064-SP)

Furthermore, navigation is more suitable for TAY and families with complex situations and/or specialized care needs. One participant described instances where navigation would be important to support transitions and access to appropriate care:

“perhaps the parents are struggling with their own mental health, perhaps the parents are struggling with frustrations with the system and just needing a little bit more care, and a little more education to reach the right service. Or again there’s complexity with the service itself or with the need itself, and that’s where navigation really comes in…navigation will develop a pathway” (047-SP).

### 3.3 Navigation to sustain continuity of care

Navigation can provide continuous support throughout the MHA journey, even during times of service transitions. Participants described the ways in which the nature and availability of support from navigators was responsive to TAY and family needs at various points through their care trajectories. This continuity can help prepare for and minimize disruptions resulting from transitions in care. To facilitate continuous care, navigators guide TAY and families through the process rather than providing one-off solutions, as a navigation service provider participant described:

“instead of giving them a list, or giving them a name and a phone number, we are actually holding that information and supporting them. We’re holding their hands to the next step, which I think is what helps people to transition from one service to another. It’s the continuity; you’ll be there even after you’ve provided them the information for the next step” (048-SP).

A caregiver participant shared how navigators facilitated continuity of care during transitions:

“they also offer to help you connect with whatever service that is, even to make an appointment, or facilitate the referral, get information, pass on information. That was very helpful because those things can be challenging sometimes. So that was very useful, and saying that they would get back to you and knowing that they wanted to follow up. It wasn’t just like calling [information/referral/access helpline] where it’s an anonymous person that you never get to speak to again. It’s a personalized service. This is a real person with a name who will email you next week or make another appointment to talk to you” (003-F).

TAY and families can return to navigation support whenever they need to, which even further promotes continuity of care. Having a navigator who knows the details of their needs can help bridge needed supports by being a continuous touchpoint, “*that one person or service that [they] can go back to*” (029-SP), and aids TAY and families in times of care transitions. A caregiver participant shared their experience of this kind of support:

“[Navigator] had been with us since the very beginning. So when we asked her, 5 years later, for advice, she had known all the different things that we had gone through and the people that he’d seen, and the outcomes of those experiences. So she was well-equipped to give us advice on what to do. I found that really reassuring because it’s somebody who knows more than me and also has been along for the ride” (010-F).

### 3.4 Navigation to support informed care

Navigation is designed to provide credible resources and information on services, which supports TAY and families in making informed decisions. A caregiver shared their experience with receiving information: “*it gives you some comfort in that the information that you have has been vetted by someone that didn’t just find it through [online searching]*” (023-F). Another caregiver shared a similar sentiment: “*I describe it as a concierge service for navigating the mental health system. They were able to explain simply the different therapies that are out there, how to access them. They did a lot of the leg work*” (008-F). The expertise of the navigator is beneficial for TAY and families who feel lost and overwhelmed in the MHA system. One TAY explained how navigation helped make them aware of available services: “*They give [you] the resources available. They help [with] finding resources that I previously might have not known about, that they exist, or that my doctor may have not mentioned. That’s probably the biggest help*” (068-TAY). Navigators maintain comprehensive knowledge and understanding of the MHA system and can thus provide care options that are tailored to meet the unique needs of each individual TAY and their families. One TAY expressed the importance of being able to rely on this expertise, stating that a navigator:

“would [say] ‘okay if this didn’t work out, I have other options for you’ and the options may not be on the [online search] list that comes up. They might have other resources that could be tailored towards my need. They would know I need something that I wouldn’t even know or recognize myself” (066-TAY).

Furthermore, navigation enables TAY and families to exercise choice in their own care. Navigators value TAY and families’ unique perspectives of their particular needs and encourage them to take the lead on their journeys. One navigator indicated, “*we see the client and the family as having something to offer, in terms of the goals that we’re developing. So they are helping to create their pathway, and we’re helping to create what’s going to happen next*” (I047-V). This also includes learning about clients’ individual barriers to care and providing information and care options in a manner that mitigates these barriers, for example, through providing navigation support in a culturally safe and responsive manner as well as care options that are culturally relevant. To better equip TAY and families in finding appropriate help, navigators also provide guidance and psychoeducation, which includes information about MHA, different types of MHA services and what to expect from them, differences between the child and adult MHA systems, MHA legislation, and language that can be helpful when engaging with other service providers. A navigator participant described the development of TAY and families’ capacity to enact informed choice in their care, by:

“Educating, supporting, just bringing them to a place to see that they have capacities…And with confidence, because they have resources, information, supports, know what to say and do” (027-SP).

Another service provider contrasted navigation support of informed choice with other centralized access services or helplines, indicating that navigators are heavily involved in providing information about:

“what to expect in terms of the treatment or the services that they are accessing, and then continuously follow up to make sure the information, wait times, eligibility hasn’t changed and the person actually gets connected. [So that it] actually feels like they understand what [TAY] are signing up for and what they are going to be getting out of the treatment or service and then the family does too and they know what’s next” (041-SP).

### 3.5 Navigation to facilitate TAY and family involvement

Navigators adopt a person-centered approach to care. Navigation “*takes the time to actually get to know the youth’s individual needs and the family’s individual needs and creates a plan based on that information, and it’s not just a generic list*” (048-SP). Another participant shared their experience engaging with a navigator who actively involved the TAY and caregiver: “*they listened to exactly, specifically what my daughter’s various issues were and what was important to her, and linked us up with someone who seems to be a really good fit*” (004-F).

Navigation can also reduce caregiver strain by lifting some of the burden off families who may feel overwhelmed from navigating the multitude of transitions encountered in TAY MHA care. This helps safeguard caregiver wellbeing, as one participant described,

“being a parent, you get the brunt of the abuse and the frustration. I think having that sort of liaison, that can help sort of keep that momentum going and encourage the patient to continue to take advantage of the resources that are offered to them” (058-F).

Another participant shared the benefits conveyed by families:

“I’ve had a lot of families tell me over and over again, ‘it’s not only the services and supports you provided to me, but it’s the relationship we developed and the confidence you gave to me to move forward each day.’ So it is that relationship-building and although we’re not providing therapy, there’s so much of a therapeutic element that goes to navigating and giving back hope and confidence [so] that they can get through the next day” (027-SP).

Navigators also support the family unit, and can provide resource options for other family members who may be in need of support. One TAY participant described the importance of this approach:

“I really liked it because they were offering even for my sister, and there is still some support for the family, which I think was helpful for my mother and myself. Before COVID we were going every now and then to see a social worker recommended by our navigator. I was going there for support for my mom because it was her first time talking to somebody about this stuff, it was just about how my mom feels…but that’s what I like about [navigation], it was not just about the person who needs the help, it was about everybody” (066-TAY).

Moreover, navigators can provide emotional support and/or validation to TAY and families, helping TAY and families feel less burdened by the responsibility of finding and connecting with MHA services. A caregiver participant intimated that their navigator’s “*validation of the struggle was hugely helpful*” (003-F). Another caregiver shared, “*it was just really very supportive, very empathetic, helpful to me, just to have somebody, just validation for me. And then also feeling like I was talking to somebody who was giving me credible advice*” (010-F).

Beyond the aforementioned TAY and family involvement and emotional support, Navigation services also provided a welcoming and comforting support in times of transitions. One TAY participant stated, “*they provide a comfort and a service that can help young adults going through a time where they are not sure of what kind of services they can get*” (050-TAY). In this way, navigation services help ensure TAY and families feel supported throughout their care journeys, as one participant concluded, “*just knowing that they’re not alone is a big deal for most people*” (048-SP).

## 4 Discussion

This study explored the role of navigation services in supporting transitions in care for TAY with MHA concerns and their families. TAY, family members, and service providers identified the ways in which navigation can help: *traverse difficult pathways*, *ensure appropriate and comprehensive care*, *sustain continuity of care*, *support informed care*, and *facilitate TAY and family involvement*. These findings emphasize the importance of tailored support for appropriate integration and navigation of care along the entire trajectory and the care transitions entailed.

Navigation was found in this study to contain elements that directly responded to the identified needs TAY, families, and providers expressed for effective transitions in Part 1 of this work.^6^ Navigation has been identified as an important support for successful care transitions in other areas of healthcare, such as dementia,21., 22. autism,23., 24. cancer,^25^ and other chronic illnesses.^26^ The ways in which navigation was identified in this study to support transitions also aligned with those previously identified as components of successful transitions, such as communication, collaboration, involvement of TAY and families, tailoring services to TAY unique needs, addressing the social determinants of health, supportive relationships, etc.27., 28., 29., 30. While there is some evidence in previous work that TAY prefer independently seeking information online,^31^ this contrasts with the findings in part 1 of this study that TAY experience a sense of overwhelm at the information available, concern about the credibility of resources, and need for guidance in understanding the information available.^6^ This study highlighted the ways in which navigation addresses these needs and can be a critical support for educating TAY and families about MHA care, as well as empowering them to make informed decisions about their care.

Moreover, it is important to consider the significance of youth perspectives in the design and implementation of MHA services and programs aimed at transitions, in order to create responsive and effective programs.32., 33., 34. While part 1 and 2 of this study highlighted the role of TAY involvement at the level of individual care, this work did not explore perspectives of youth involvement at the service or system level. However, it is known from other work that youth engagement can occur at various levels in MHA navigation services and that youth becoming involved in an advisory capacity in MHA navigation services are seeking to make an impact on the system.35., 36. Furthermore, youth peer roles have been explored in navigation services outside of MHA care,37., 38., 39. and thus can hold promise in youth MHA navigation services as well. Future work should explore the mechanisms by which this can best be achieved, to ensure that navigation services are co-designed in a manner that respond to TAY and family needs during transitions in care.

Although many TAY and families had been connected to various services in the MHA system, they identified difficulties transitioning to the next service, be these age-related transitions or otherwise. These experiences emphasized that no matter the availability of services and level of integration of the system, there will always be some need for navigation. Other roles have been described to help bridge gaps and assume responsibility for transitions, such as transition workers,^40^ transition coordinators, case managers, etc.;^41^ these roles include system navigation as a component of their work but often do not declare navigation as the primary function of their work. Information and resource referral/helpline and coordinated access models are also gaining traction in the MHA system (e.g., AccessMHA, ConnexOntario) and are extremely beneficial for many TAY and families.42., 43. However, these may not provide the level of individualized information and continued supportive guidance desired by TAY and families in transition and provided by navigation services, that was identified in this study. MHA navigation services with varying models are also becoming more readily available. For example, they can be targeted at TAY and families directly (e.g., FNP),^44^ can support primary care physicians in directing their own patients to appropriate care (e.g., SCOPE),^45^ or can be embedded within broader MHA services (e.g., Canadian Mental Health Association navigator programs),46., 47. to name a few. Future work can specifically explore the role of navigation services in building connections and promoting collaboration and continuity of care among MHA services as well as between MHA services and primary care, as well as the outcomes of these activities.

Finally, TAY and families can experience multiple crisis situations in times of transition, where they may feel failed by the MHA system in the time leading up to and following the crisis.^6^ Cohen and colleagues described youth’s engagement in services during times of crisis, often in clusters of multiple services in short periods of time.^48^ Unfortunately, youth often do not receive ongoing support during or after their engagement with crisis services.^6^ Navigation has the potential to reduce this crisis-driven engagement, by acting as a touchpoint in the system during times of crisis and related transitions, and by enhancing access to and continuity of care. While some preliminary work has explored navigation for the purposes of emergency department diversion,^49^ future work can explore the impact of navigation services on the use of crisis supports specifically among TAY with MHA concerns. Ultimately, TAY and families can benefit most from a transition support approach, such as youth MHA navigation, that knowledgeably takes advantage of the full range and extent of services available throughout the entirety of the MHA system. In light of these findings, service providers supporting TAY who are facing upcoming transitions may consider connecting the TAY with a navigation service to provide needed support and guidance for the TAY and family through the care trajectory. In these cases, service providers (e.g., youth workers, case workers, support group facilitators, etc.) can remain involved with the TAY and family alongside the navigation service if they are willing and able to do so. Decision-makers may wish to implement youth MHA navigation services in their jurisdictions, to support TAY and families during critical transition junctures and support the integrated, coordinated, and continuous care across their MHA systems.

### 4.1 Limitations

Participants, particularly TAY and families, had a range of experiences with navigation services, from none at all to extensive support from a navigation service. Findings may therefore over- or under-represent the role of navigation in supporting transitions in care for TAY with MHA concerns and their families. Future work may specifically explore the effectiveness of care transitions for TAY and families with and without experiences of navigation, to ascertain the impact of navigation supports. Although study recruitment encouraged involvement from participants with diverse perspectives, certain groups are not represented, such as foster youth and families, Indigenous youth and families, etc. Thus, further research is needed to specifically explore the perspectives of participants with more diverse backgrounds and experiences. Furthermore, although the original study plan included interviews with system decision-makers (e.g., directors of TAY MHA services, policy-makers, etc.), data collection coincided with the onset of the COVID-19 pandemic. Individuals in decision-maker roles rapidly became unavailable as they were required to develop and implement protocols and plans for their services. Thus, decision-maker perspectives are notably not present in the findings. Future work may consider focusing on decision-maker perspectives regarding the role of navigation to support transitions in care for TAY with MHA concerns, to inform the scale and spread of navigation services for this purpose.

### 4.2 Conclusions

Navigation supports are a meaningful way to alleviate challenges and address needs associated with transitions in care, particularly for TAY with MHA concerns and their families. Navigation can assist TAY with MHA concerns and their families in effectively finding their way through transitions along their care pathways and accessing care that is appropriate to their needs, all the while feeling engaged and empowered in making decisions that meet their unique needs. Given the inevitability of multiple transitions in MHA care journeys, navigation has the potential to play a significant role in enhancing access to and transitions in care for TAY and their families, and to enrich a transformation of the MHA system at large.

## Ethics statement

All study procedures were performed in compliance with relevant laws and institutional guidelines and have been approved by the Sunnybrook Health Sciences Centre Research Ethics Board (#339–2019, October, 2019). Informed consent was obtained for research with human participants.

## Funding statement

Financial support for the conduct of the research was provided by the 10.13039/501100000024Canadian Institutes of Health Research (Funding Reference Number: TEG165589). The funder had no role in study design; collection, analysis, and interpretation of data; in writing the report; or in the decision to submit the article for publication.

## CRediT authorship contribution statement

**Deepy Sur:** Writing – review & editing, Validation, Methodology, Investigation, Funding acquisition. **Amy Cheung:** Writing – review & editing, Validation, Methodology, Investigation, Funding acquisition. **Jocelyn Charles:** Writing – review & editing, Validation, Methodology, Investigation, Funding acquisition. **Cathy Walsh:** Writing – review & editing, Validation, Methodology, Investigation, Funding acquisition. **Tracey Addison:** Writing – review & editing, Validation, Methodology, Investigation, Funding acquisition. **Sugy Kodeeswaran:** Writing – review & editing, Validation, Resources, Project administration, Methodology, Investigation, Funding acquisition, Conceptualization. **Karen Wong:** Writing – review & editing, Validation, Formal analysis. **Hinaya Cader:** Writing – review & editing, Writing – original draft, Validation, Formal analysis, Data curation. **Roula Markoulakis:** Writing – review & editing, Writing – original draft, Validation, Supervision, Resources, Project administration, Methodology, Investigation, Funding acquisition, Formal analysis, Data curation, Conceptualization. **Anthony Levitt:** Writing – review & editing, Validation, Supervision, Resources, Project administration, Methodology, Investigation, Funding acquisition, Conceptualization. **David Willis:** Funding acquisition, Investigation, Methodology, Validation, Writing – review & editing.

## Declaration of Competing Interest

The authors declare that they have no known competing financial interests or personal relationships that could have appeared to influence the work reported in this paper.

## Data Availability

Data will be made available on request.
